# Development of Conjugated Polymers for Memory Device Applications

**DOI:** 10.3390/polym9010025

**Published:** 2017-01-12

**Authors:** Hung-Ju Yen, Changsheng Shan, Leeyih Wang, Ping Xu, Ming Zhou, Hsing-Lin Wang

**Affiliations:** 1 Physical Chemistry and Applied Spectroscopy (C-PCS), Chemistry Division, Los Alamos National Laboratory, Los Alamos, NM 87545, USA; csshan720@gmail.com; 2 Center for Condensed Matter Science, National Taiwan University, 1 Roosevelt Road, 4th Sec., Taipei 10617, Taiwan; leewang@ntu.edu.tw; 3 School of Chemistry and Chemical Engineering, Harbin Institute of Technology, Harbin 150001, China; pxu@hit.edu.cn; 4 Department of Chemistry, Northeast Normal University, Changchun 130024, China; zhoum739@nenu.edu.cn

**Keywords:** conjugated polymer, memory device, dynamic random access memory (DRAM), static random access memory (SRAM), write-once read-many-times (WORM), flash

## Abstract

This review summarizes the most widely used mechanisms in memory devices based on conjugated polymers, such as charge transfer, space charge traps, and filament conduction. In addition, recent studies of conjugated polymers for memory device applications are also reviewed, discussed, and differentiated based on the mechanisms and structural design. Moreover, the electrical conditions of conjugated polymers can be further fine-tuned by careful design and synthesis based on the switching mechanisms. The review also emphasizes and demonstrates the structure-memory properties relationship of donor-acceptor conjugated polymers for advanced memory device applications.

## 1. Introduction

### 1.1. Conjugated Polymers (CPs)

**Scheme 1:**
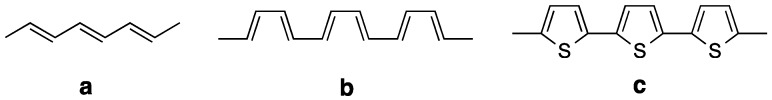
Structures of conjugated polymers (**a**) trans- and (**b**) cis-polyacetylene; and (**c**) polythiophene.

Conjugated polymers (CPs) are organic macromolecules characterized by a backbone chain consisting of alternating single- and double-bonds. Their overlapping *p*-orbitals create a delocalization system of π-electrons thus resulting in interesting and useful optoelectronic properties. The simplest CP, polyacetylene, constitutes the core of all conjugated polymers although it is itself too unstable for any practical applications (Scheme 1). Owing to its structural and electronic simplicity, polyacetylene is well suited to ab initio and semi-empirical calculations, which has played a critical role in the theoretical aspects of CPs [1].

**Scheme 2:**
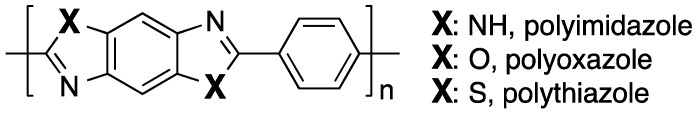
Structures of typical rigid rod polybenzobisazoles.

Attention on π-CPs has increased over the years [2,3,4,5,6]. The extensive delocalization of π electrons in CPs is well recognized to be responsible for their remarkable characteristics, including electronic conductivity, interesting optical characteristics, and exceptional mechanical properties [7,8] such as high tensile strength and resistance to harsh environments. CPs comprising aromatic or hetero-aromatic ring structures have also been considered particularly as outstanding materials. Moreover, applications of CPs in advanced aerospace technology have provided a powerful motivation for the development of functionalized polyhetero-aromatics [9]. Later investigations were directed to rigid rod polybenzobisazoles (Scheme 2).

**Scheme 3:**
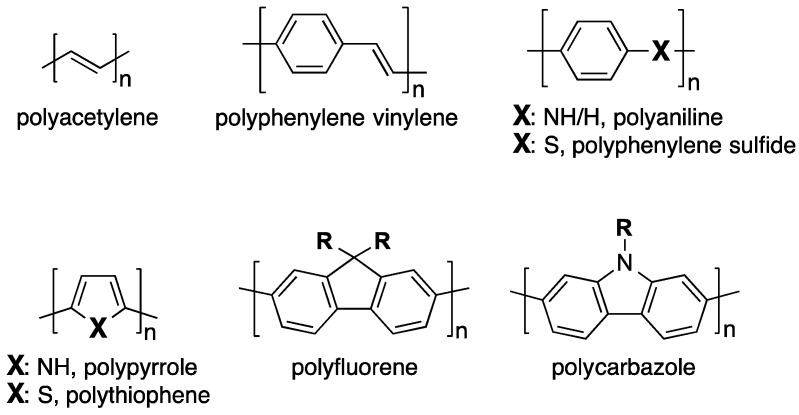
Structures of conventional conjugated polymers.

From an electrochemical perspective, the most important aspect of CPs is their capability as electronic conductors. Therefore, π-electron polymers have been the focus of extensive research [10], ranging from applications of conventional CPs (as shown in Scheme 3) in energy storage devices, to novel CPs with special electronic properties such as low band-gap and intrinsic conductivity. Indeed, many successful commercial applications of CPs have been available for more than fifteen years, including capacitors, batteries, magnetic storage, electrostatic loudspeakers, and anti-static bags.

Recently, CPs have been considered as electroactive memory materials and reported as revealing electrically volatile and non-volatile memory characteristics. The conjugated backbone is the most important component since it contributes to most of the electrical properties. In general, the incorporation of different electron acceptors into CPs significantly affects the memory properties, which can either create a trapping site or provide the charge transfer (CT) conducting channel. In addition, the stability of charge trapping or the CT complex process, further determines the volatility of the memory device.

### 1.2. Resistor-Type Electronic Memories

The basic goal of a memory device is to provide a means for storing and accessing binary digital data sequences of “1” and “0”, being one of the core functions (primary storage) of modern computers. An electronic memory is a form of semiconductor storage, fast in response, compact in size, and can be read and written when coupled with a central processing unit (CPU). In the conventional silicon-based electronic memory, data are stored based on the amount of charge stored in the memory cells. On the contrary, organic/polymer electronic memory stores data in an entirely different way, for instance, based on the different electrical conductivity states (ON and OFF states) in response to the applied electric field. In particular, polymeric memory devices with an electrically bi-stable behavior have received considerable attention recently due to their attractive characteristics such as rich structure flexibility, low cost, solution processability, and three-dimensional stacking capability. Therefore, the organic/polymer electronic memory is likely to be alternative or supplementary to the conventional semiconductor electronic memory.

**Figure 1:**
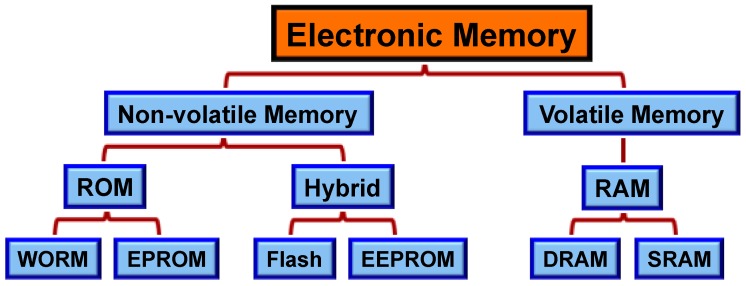
Classification of electronic memories. ROM: read-only memory; RAM: random-access memory; EPROM: erasable programmable read-only memory; EEPROM: electrically erasable programmable read-only memory.

Electronic memories can be generally divided into two primary categories according to the storage type: volatile and non-volatile memories (Figure 1). Volatile memory eventually loses the stored information unless it is provided with a constant power supply or refreshed periodically with a pulse; non-volatile memory is capable of holding data permanently and being read repeatedly. Among these types of electronic memories, write-once read-many-times (WORM) memory [11], hybrid non-volatile and rewritable (flash) memory [12], static random access memory (SRAM) and dynamic random access memory (DRAM) are the most widely reported polymer memories [13,14].

Memory devices incorporating switchable resistive materials are generally classified as resistor-type memory, or resistive random access memory (RRAM). Unlike transistor and capacitor memories, a resistor-type memory does not require a specific cell structure (e.g., field-effect transistor; FET) or to be integrated with the complementary metal-oxide-semiconductor (CMOS) technology. The electrical bi-stability of resistor-type memories usually results from the changes in intrinsic properties of electroactive materials in response to the applied voltage or electric field, such as charge transfer, phase change, conformation change, and redox reaction [15].

The important parameters to the memory performance include switching (write and erase) time, ON/OFF current ratio, read cycles, and retention time. The switching time influences the rate to write and access the stored information; the ON/OFF current ratio defines the control of the misreading rate during device operation; while the number of read cycles and long retention time are related to the stability and reliability of the memory devices. For practical applications, other factors, such as power consumption and cost, structural simplicity and packing density, as well as mechanical stiffness and flexibility, are of equal importance when designing and fabricating new memory devices. Considerable efforts have been devoted to develop novel CPs for information and communication technologies [16,17,18,19,20,21].

#### 1.2.1. Operation Mechanism

Many research works have been dedicated to understanding the electric switching phenomena of memory devices. Although this field is still controversial, researchers have proposed several well established switching mechanisms based on theoretical simulations, experimental results, and advanced analytical techniques [15,22,23,24,25,26,27]. In this review, we summarize the most widely used mechanisms in CP resistive memory devices, such as charge transfer, space charge traps, and filamentary conduction.

##### Charge Transfer (CT)

CT can be clarified as a process of partial transfer of electronic charge from the donor (D) to the acceptor (A) moiety in the electron D-A system by applying a suitable voltage, which can result in a sharp increase in conductivity [28]. In order to obtain a better understanding of switching mechanisms, several study methods, such as density functional theory (DFT) calculations, ultraviolet-visible (UV/Vis) absorption spectra, in-situ fluorescence spectra, and transmission electron microscope (TEM) techniques, can be used to investigate and explain the CT phenomenon [29,30,31,32]. CT is anticipated to occur most frequently in D-A polymers [33,34,35]. The memory behaviors based on the D-A polymers can be tuned adequately through modification of the polymer structures. By tuning the electron-donating or -accepting capability of D-A polymers, different memory behaviors can be achieved [36]. The strong dipole moment in a polymer is also beneficial to maintain the conductive CT state, usually leading to non-volatile memory behavior. Otherwise, the conductive CT state is not stable after removing the electric field and a volatile memory characteristic will be observed if the dipole moment is not strong enough.

##### Space Charge Traps

When the interface between electrode and polymer is ohmic and the polymer is trap-free, the carriers near the electrode will accumulate and build up a space charge channel. Mutual repulsion between individual charges restricts the total charge injected into the polymer, and the resulting current is defined as space charge-limited current (SCLC). Space charges in materials may occur from several sources, such as (1) electrode injection of electrons and/or holes; (2) ionized dopants in interfacial depletion regions; and (3) accumulation of mobile ions at electrode interfaces. Traps may be present in the bulk materials or at interfaces, and result in lower carrier mobility. When present at interfaces, they may also affect charge injection into the materials. The electrical switching behaviors of some polymeric materials have been reported to be associated with space charges and traps [30].

##### Filament Conduction

Particularly, when the ON state current is highly localized to a small area within the memory device, the phenomenon can be termed “filament conduction”. It has been suggested that filament conduction is confined to device physical damage in RRAMs. Two types of filament conduction have been widely reported in polymer resistive memory devices, and the formed filaments could be observed under an optical microscope or scanning electron microscope [37,38]. One type is carbon-rich filaments formed by local degradation of polymer films [38,39]. The other is associated with metallic filaments that result from migration of electrodes through the polymer films [40,41]. For filamentary conduction, the polymers with both the coordinating atom and π-conjugation can bind to metal ions, regardless of the binding sites as side chain or main chain, are essential for the production of metal filaments [15,42]. Therefore, the filamentary conduction mechanism has been often suggested to explain switching phenomena observed in a variety of polymer memory devices. However, the severe current leakage caused by the filament effect is the main factor in restricting the exploration of memory mechanism. Therefore, some literature examples discussing how to reduce the filament effect have been reported [43,44]. Unfortunately, although the device concept is simple, the physics is anything but. There are controversies surrounding the conductive filament and the role of the top and the bottom electrodes. The mobility, energy, and stability of the oxygen vacancies remain topics of intense study. As a result of these open issues, projection of device reliability becomes difficult. Furthermore, the switching mechanisms in different references of similar structures are always various, and they need to be unique from both an understanding point of view as well as for application. RRAMs have issues on reproducibility of their electrical characteristics; there are large resistance variations not just between devices, but also between cycles of programming of the same device. Therefore, selecting the switching material and deposition method also plays an important role.

## 2. CPs for Volatile Memory Devices

**Figure 2:**
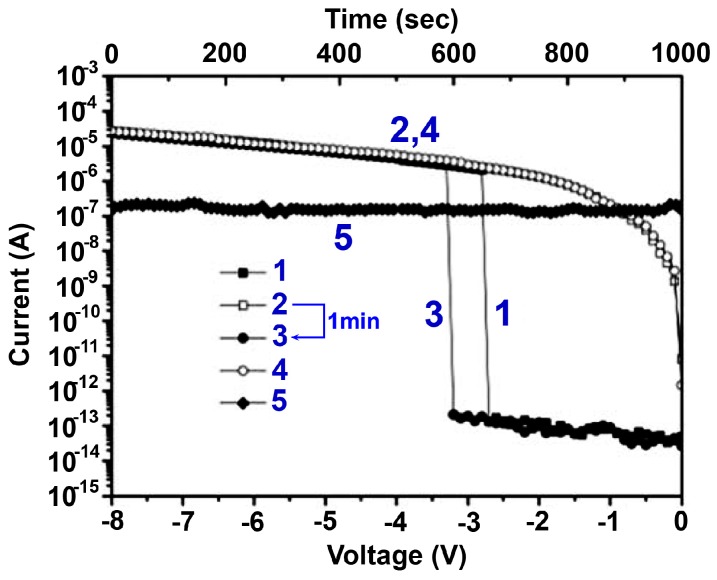
Current-voltage (I–V) characteristics of the indium tin oxide (ITO)/polymer/Al memory device as a representative of DRAM characteristic. (The third sweep was conducted about 1 min after turning off the power).

**Figure 3:**
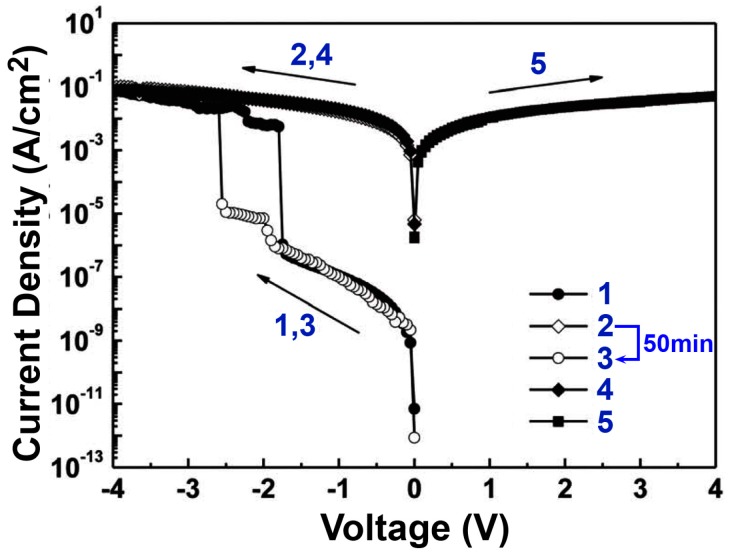
Current-voltage (I–V) characteristics of the ITO/polymer/Al memory device as a representative of SRAM characteristic. (The third sweep was conducted about 50 min after turning off the power).

For volatile memory effects, the device cannot be kept at the ON state and will relax to the OFF state after the power is turned off. Nevertheless, the ON state can be maintained by refreshing the voltage pulse. Volatile memory effects can be divided into DRAM and SRAM, depending on the retention time of the ON state after removal of the applied voltage. For DRAM behavior (Figure 2) [30], the ON state can only be retained for a short time (less than 1 min) after the removal of the applied voltage. For SRAM (Figure 3) [45], the device can stay in the ON state for a longer period of time after turning off the power than in DRAM devices. Despite the longer retention time of the ON state in SRAM memory devices, it is still volatile, and the ON state will relax to the OFF state without an erasing process.

### 2.1. Dynamic Random Access Memory (DRAM) Properties

**Scheme 4:**
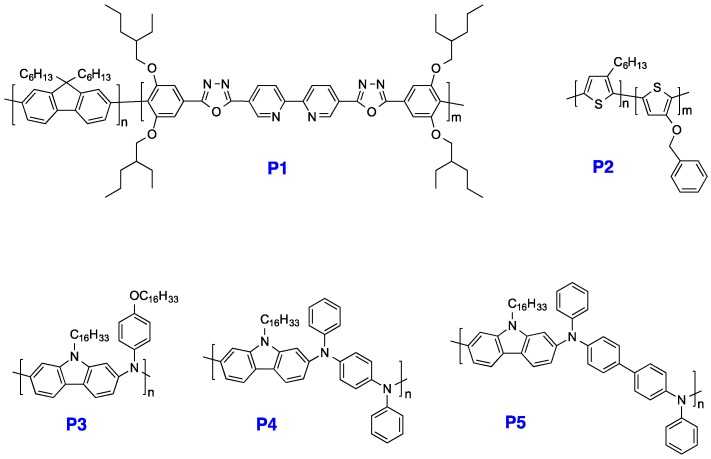
Chemical structures of some polymers with DRAM memory properties.

**P1** containing oxadiazole and bipyridine as acceptor units was synthesized by Suzuki coupling and exhibited DRAM memory behavior with an ON/OFF ratio of more than 10^6^ (Scheme 4) [14]. The memory effect was volatile due to space charge and traps, resulting in the short retention ability of its ON state. The ON state current could be electrically sustained by a refreshing voltage pulse every 10 s.

Devices with the sandwich structure of ITO/**P2**/Al exhibited volatile DRAM property with bi-stable electrical switching characteristics, which is due to the existence of trapping sites in the poly(3-phenoxymethylthiophene) domains, whereas poly(3-hexylthiophene) devices only showed semiconductor characteristics [30]. This result suggested the importance of the amorphous poly(3-phenoxymethylthiophene) segments on the electrical switching behavior. Both the ON and OFF states of **P2** are stable up to 10^8^ read cycles under a constant voltage stress of −1.0 V with a high ON/OFF current ratio of about 10^6^.

Ree et al. also investigated the memory characteristics of arylamine-linked poly(2,7-carbazole)s **P3**, **P4**, and **P5** [46]. These polymers are amorphous but slightly oriented in the film plane. All polymers with the sandwich structure of ITO/polymers/Al were found to exhibit similar DRAM behaviors without polarity. The devices are programmable at low voltage with a high ON/OFF current ratio up to 10^9^ as the thickness ranged between 8 and 60 nm. The memory behaviors are governed by SCLC and local filament formation, which might originate from the electron-donating carbazole and triphenylamine units in the polymer backbones.

### 2.2. Static Random Access Memory (SRAM) Properties

**Scheme 5:**
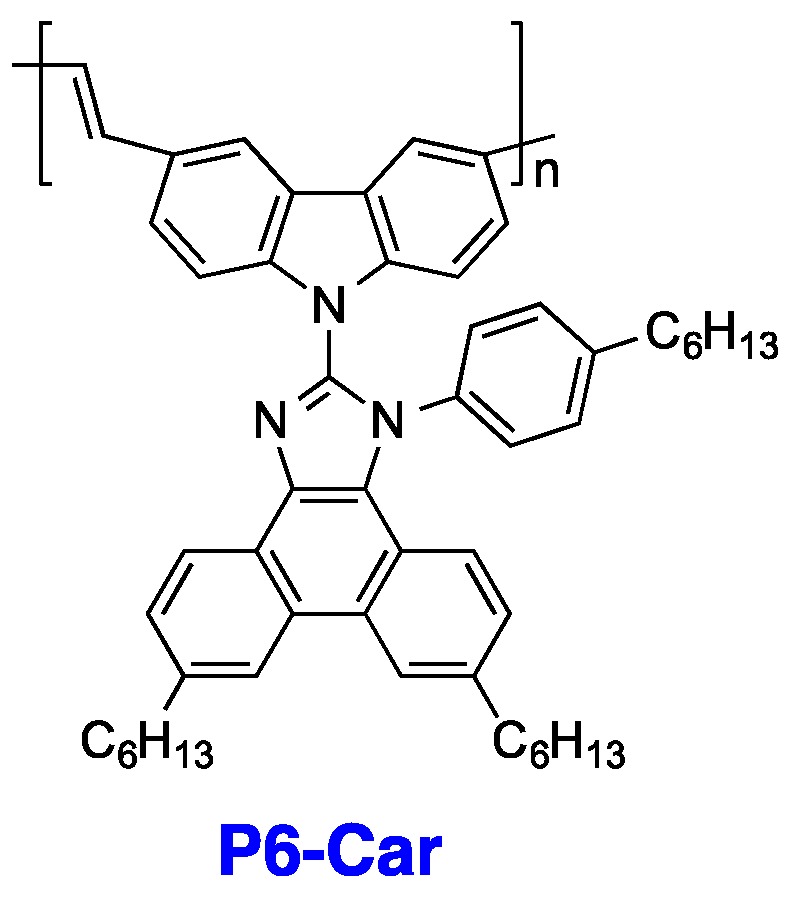
Chemical structure of polymer with SRAM memory property.

Chen et al. reported a D-A CP, poly(arylenevinylene), consisting of carbazole (Car) with pendent phenanthro[9,10-*d*]imidazole (**P6-Car**) (Scheme 5) [45]. The flexible **P6-Car** device with the sandwich configuration of poly(ethylene-2,6-naphthalate) (PEN)/Al/**P6-Car**/Al revealed volatile SRAM characteristic, which can be operated at low voltages with high ON/OFF current ratios (more than 10^4^) and excellent durability. The high steric hindrance between carbazole donor and phenanthro[9,10-*d*]imidazole side chain leads to a weak electric charge separated state and easy recombination after turning off the electrical power, resulting in volatile memory characteristics.

## 3. CPs for Non-Volatile Memory Devices

**Figure 4:**
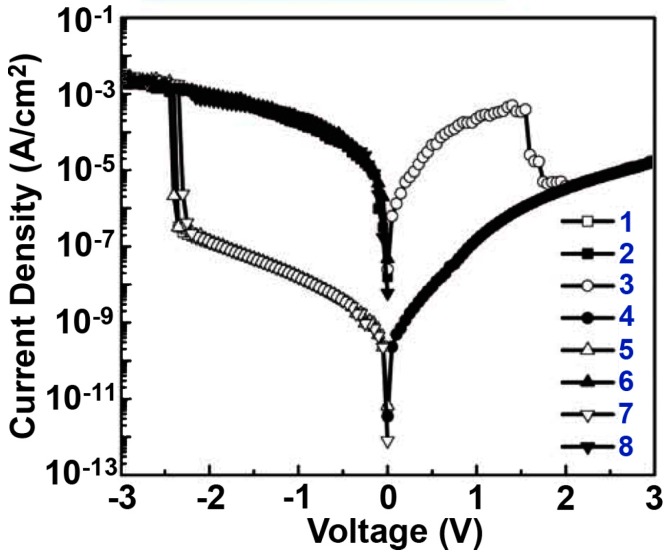
Current-voltage (I–V) characteristics of the ITO/polymer/Al memory device as a representative of Flash characteristic.

**Figure 5:**
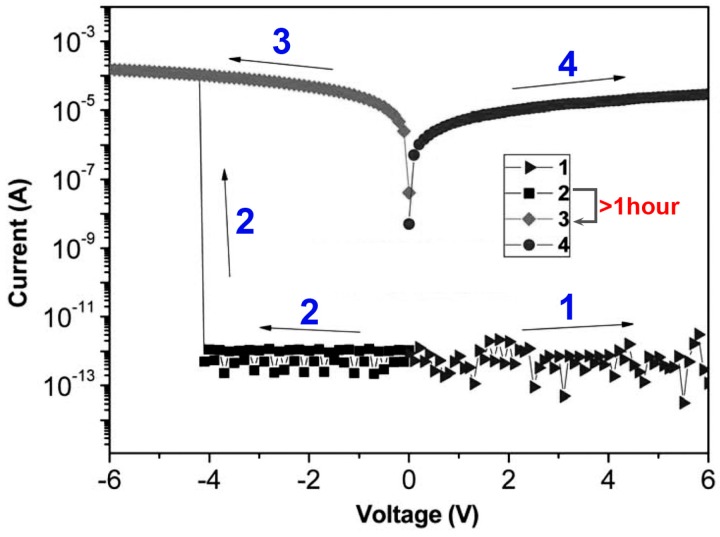
Current-voltage (I–V) characteristics of the ITO/polymer/Al memory device as a representative of WORM characteristic. (The third sweep was conducted more than one hour after turning off the power).

Non-volatile memory devices can stay in the ON state steadily without an applied voltage bias. Non-volatile memory behaviors can be divided into two classes, namely WORM memory and rewritable (flash) memory, depending on whether a suitable voltage can switch the ON state to the OFF state or not. If the ON state can be switched back to the OFF state by applying a suitable voltage, which is an erasing process, the memory effect is called rewritable memory (Figure 4) [47]. However, WORM is capable of maintaining the ON state (holding data) permanently, even applying a reverse voltage (Figure 5) [48].

### 3.1. WORM Properties

**Scheme 6:**
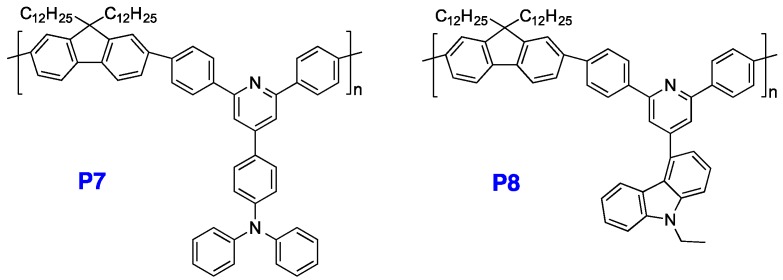
Chemical structures of some polymers with WORM memory properties.

The non-volatile memory effect was investigated in other fluorene-acceptor push-pull polymeric systems [14,49,50,51,52,53,54,55]. **P7** consisting of 9,9-didodecylfluorene, pendent triphenylamine donors, and pyridine acceptors exhibited WORM memory behavior (Scheme 6) [49]. Hole injection from ITO into the highest occupied molecular orbital (HOMO) of **P7** is an energetically favored process due to the low energy barrier between the work function of ITO and the HOMO level of **P7**. The injected hole migrates through the continuous positive electrostatic potential channel along the polymer chain and becomes trapped by the electron acceptor group (nitrogen atom in the pyridine ring) but cannot get activated by a reverse voltage bias, resulting in the WORM type memory behavior.

Nonvolatile WORM memory behavior with tristable property was obtained by poly(2,6-diphenyl-4-((9-ethyl)-*9H*-carbazole)-pyridinyl-*alt*-2,7-(9,9-didodecyl)-*9H*-fluorenyl) (**P8**) [51]. The device switched from the initial low-conductivity (OFF) state to the first high-conductivity (ON-1) state at a threshold voltage of 1.8 V, and subsequently to the second high-conductivity (ON-2) state at a higher voltage of 2.4 V, which is also elucidated by the enhanced UV-Vis absorption. Under an applied field, the process of conformational order, arising from charge carrier delocalization-induced D-A interaction to form a partial or complete face-to-face conformation of the fluorene and carbazole units, can be generated throughout the polymer layer. An effective charge transport channel originated from the electron hopping between the ordered structures effectively switched the device from the OFF to ON-1 state. The coordinating ability of the nitrogen atoms in the carbazole pendant moieties and the indium atoms of ITO also promoted the charge transfer at the polymer/ITO interface.

### 3.2. Flash Properties

**Scheme 7:**
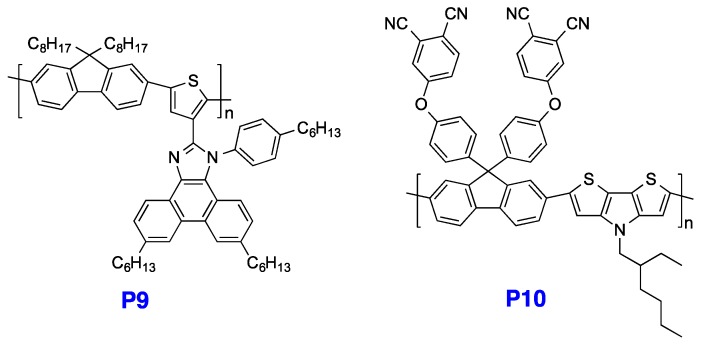
Chemical structures of some polymers with Flash memory properties.

Chen et al. reported a flexible bipolar resistive memory device with reliable performance in response to electric and mechanical stimuli by using a conjugated poly(fluorene-thiophene) donor tethered phenanthro[9,10-*d*]imidazole acceptor (**P9**) as the active layer (Scheme 7) [54]. The **P9** device exhibited low threshold voltages, large ON/OFF memory windows, and good retention time. In the backbone of **P9**, fluorene and thiophene donors act as hole transporters/trapping centers, while the phenanthro[9,10-*d*]-imidazole acceptor serves as an electron transporter/trapping center. Both the donor and acceptor act as trapping sites that depend on the charge association and polarity of the electric field. As the applied voltage approaches the threshold voltage, the majority of trapped charges are filled to create a trap-free environment. The captured charges and the charged states can be maintained due to a large energy barrier for the back transfer of charges, and the deep trapping sites may not be easily recovered even after turning off the power. However, under a reverse voltage bias, the trapped charges can be extracted and then return the device back to the OFF state, leading to bipolar flash-type memory behavior. Chen and Li designed a fluorene-acceptor copolymer **P10** for resistor memory where 9,9-bis[4-(4-phenoxyl)phthalonitrile] pendant groups at the C-9 position of the fluorene unit are electron acceptors [55]. A strong dipole moment in **P10** (10.71 Debye) is also beneficial for maintaining the conductive state.

### 3.3. Negative Differential Resistance (NDR) Properties

**Scheme 8:**
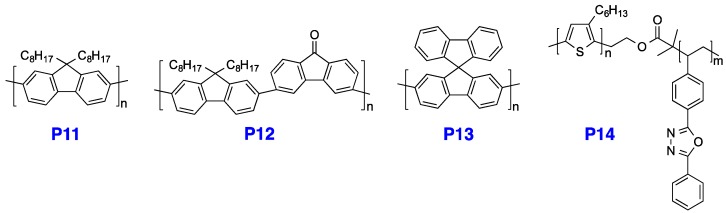
Chemical structures of some polymers with negative differential resistance (NDR) memory properties.

Non-volatile resistive memory properties based on polyfluorenes have been statically characterized [56,57]. The working mechanism was attributed to metallic filaments with a write pulse at 4 V and an erase pulse ranging from 8–10 V. Lee et al. used two different polyfluorenes, **P11** and **P12**, and two Al and Au electrodes to fabricate the memory devices, respectively (Scheme 8) [58]. Bi-stability was observed in all devices with deposited Al as electrode. On the contrary, bi-stable switching was observed only when Au was deposited on the oxidized polyfluorene **P12**. Presumably, both the internal trap site and organic/metal interface were responsible for the electric bi-stability of these memory devices. Gomes’ group also reported resistive memory devices based on poly(spirofluorene) **P13** using small signal impedance measurements [59,60]. The device remained highly resistive but the low frequency capacitance increased by several orders of magnitude. Higher external applied voltages led to an increased electrical stress across the oxide, which reduced the resistance, hence the switching.

**Figure 6:**
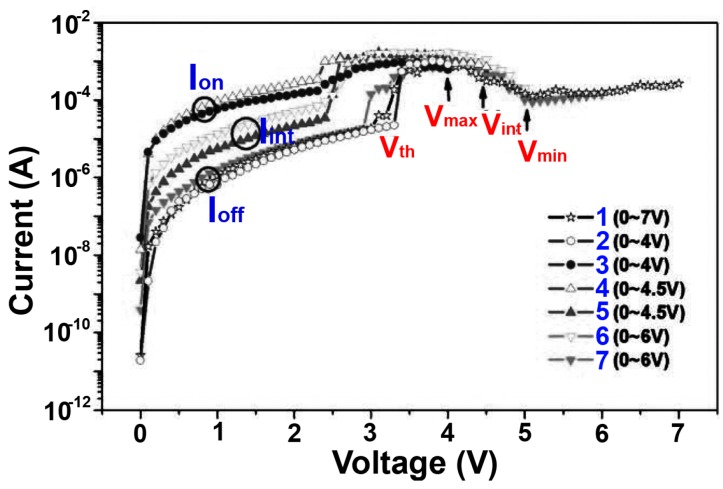
Current-voltage (I–V) characteristics of the ITO/polymer/Al memory device as a representative of NDR characteristic.

Chen et al. have reported the synthesis of novel D-A rod-coil diblock copolymer **P14** and its memory device [61]. The highest occupied molecular orbital (HOMO) energy level at −6.08 eV for polyoxadiazole was employed as a charge trap for electrical switching memory devices. The ITO/**P14**/Al memory device exhibited nonvolatile memory property with a NDR effect due to the polyoxadiazole charge trapped block (Figure 6).

## 4. Effects of the Molecular Design on Volatility

### 4.1. Donor Effect

**Scheme 9:**
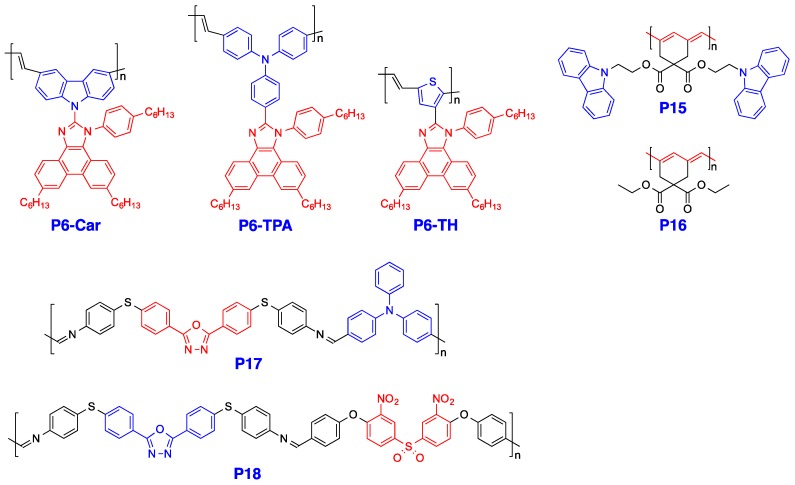
Chemical structures of some polymers with donor effect.

The tunable electrical switching characteristics of the vinylene-based CPs [47], **P6-Car**, **P6-TH**, and **P6-TPA**, consisting of various donors, such as carbazole, thiophene, and triphenylamine, respectively, with the pendant acceptor of phenanthro[9,10-*d*]imidazole were demonstrated (Scheme 9). The donor structures not only affected the polymer conformation, but also the D-A interaction and LUMO energy levels for stabilizing the charge separation. The PEN/Al/**P6-Car**/Al flexible device revealed SRAM behavior while the **P6-TPA** device exhibited WORM property, both memory devices can be operated at low voltages with high ON/OFF current ratios and excellent durability under repeated bending tests. However, the **P6-TH** device only exhibited a diode-like electrical behavior.

Ree et al. reported the programmable memory characteristics of fully π-conjugated polymers **P15** and **P16** [62,63]. The memory properties of **P15** were investigated as a function of temperature and film thickness. **P15** with a thickness of 15–30 nm showed excellent unipolar DRAM behavior with a high ON/OFF ratio up to 10^8^, which was mainly governed by filament formation supported by the metallic properties of the **P15** film. The ON state current was dominated by Ohmic conduction, and the OFF state current appeared to undergo a transition from Ohmic to space charge limited conduction with a shallow-trap distribution. On the other hand, **P16** with a thickness of 30 nm exhibited a very stable WORM memory property with an ON/OFF ratio of 10^6^. Both the ester units and the conjugated double bonds of the **P16** polymer backbone acted as efficient charge trapping sites.

Li et al. also reported two donor-acceptor type poly(azomethine)s, incorporating an oxadiazole group either acting as an electron acceptor in **P17** with the triphenylamine donor, or serving as a donor in **P18** with the 3,3′-dinitro-diphenylsulfone acceptor [64]. The variation in the role of the oxadiazole group in the D-A polymers resulted in different memory properties of the prepared poly(azomethine)s. The **P17**-based memory device with Pt/**P17**/Pt sandwiched structure showed rewritable memory behavior but poor endurance of less than 20 cycles, while the **P18**-based device exhibited WORM memory behavior. The different memory properties are attributed to the different band gaps of the poly(azomethine)s, indicating the different degree of intra- and intermolecular charge transfer interaction between the electron donor and acceptor. The stronger electron push-pull interaction in **P17** facilitates the charge transfer effect, resulting in a lower switching threshold voltage than that of the **P18**-based device. For the memory devices with Al/polymer/Al sandwiched structure, both **P17** and **P18** demonstrate a much improved resistive switching effect, and the endurance of the **P18**-based device is better than that of the **P17**-based device. The difference in the electronic transport and memory properties of the four devices may originate from the different charge injection/extraction and electron transfer processes of the sandwich systems.

### 4.2. Acceptor Effect

**Scheme 10:**
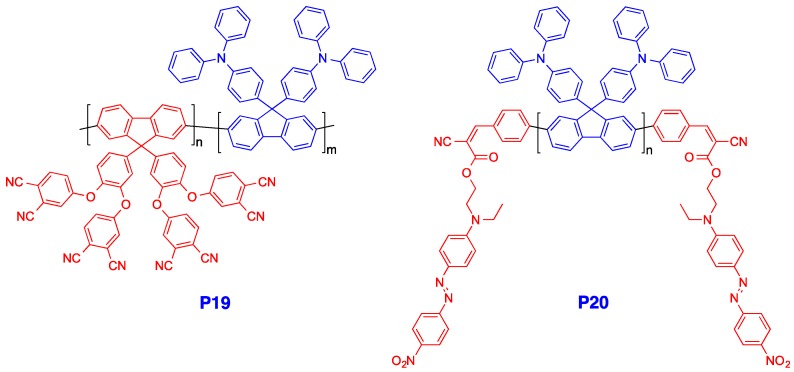
Chemical structures of some polymers with acceptor effect.

Polyfluorene-based copolymer **P19** containing electron-donor triphenylamine and electron-acceptor 9,9-bis[3,4-bis(3,4-dicyanophenoxy)phenyl] side chains at the C-9 position of the fluorene unit was applied for a nonvolatile WORM memory device (Scheme 10) [52], meanwhile **P20** consisting of poly[9,9-bis(4-diphenylaminophenyl)-2,7-fluorene] donors with end-capped Disperse Red 1 exhibited bi-stable conductive states and rewritable memory behavior [50]. Under a low positive voltage sweep, favorable hole injection and migration resulted in the formation of a high current state. As the hole injection process was underway, the positive charges on the triphenylamine moieties in **P19** were rapidly consumed by the cyano groups as a result of the irreversible switching operation. On the contrary, the active area- or temperature-independent current density in **P20** indicated the absence of sample degradation or breakdown and excluded the metallic filamentary conduction effect.

### 4.3. Thickness Effect

**Scheme 11:**
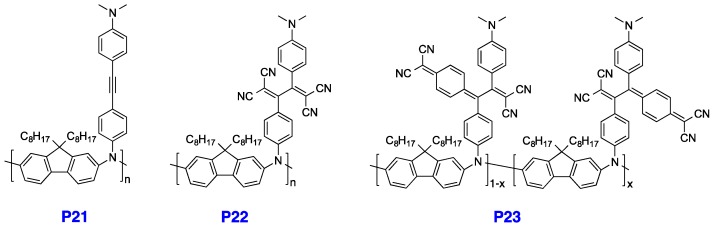
Chemical structures of some polymers with thickness effect.

Ree et al. reported donor-acceptor polymers, **P21**, **P22,** and **P23**, which were composed of fluorene, triphenylamine, dimethylphenylamine, alkyne, tetracyanoethylene (TCNE), and 7,7,8,8-tetracyanoquinodimethane (TCNQ) adducts (Scheme 11) [65]. The TCNE and TCNQ units were found to enhance the π-conjugation lengths and intramolecular charge transfer of **P22** and **P23**, respectively, despite their electron-acceptor characteristics. The TCNE and TCNQ units enabled the authors to fine-tune the memory properties and widen the thickness window of the polymer layer. In the memory device with Al/polymer/Al sandwiched structure, **P21** exhibited stable unipolar permanent memory behavior with high reliability. On the other hand, **P22** and **P23** devices showed stable unipolar permanent memory behavior over only a narrow film thickness window of 10–20 nm while DRAM behavior can be obtained with a wider thickness window of 10–30 nm at higher operation voltages. The memory behavior of **P23** was observed to be driven by both hole and electron injection in which the electron donor and acceptor groups both acted as charge trapping sites, indicating that the memory devices can be operated at relatively low voltages.

## 5. CPs Containing Metal Complexes

**Scheme 12:**
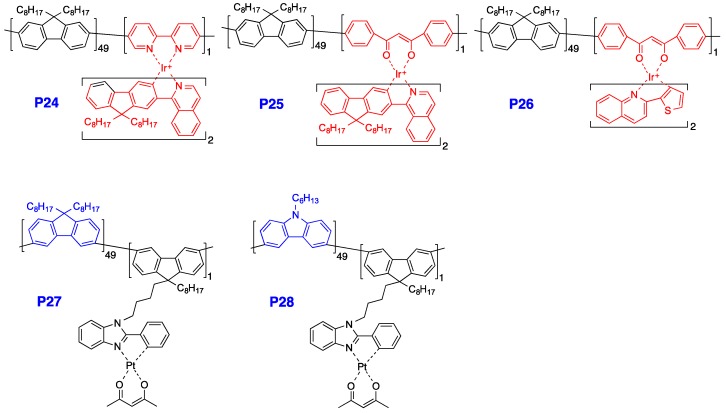
Chemical structures of some polymers containing metal complexes.

Conjugated polyfluorenes with cationic Ir(III) complexes, **P24**–**26**, were selected as active memory materials for the functionalities of flash memory devices [33,66]. The memory device based on **P24** containing Ir(III) complex exhibited low reading, writing, and erasing voltages with a high ON/OFF current ratio of more than 10^5^ (Scheme 12). Both ON and OFF states were stable under a constant voltage stress of −1.0 V up to 10^8^ read cycles. The flash memory behavior was attributed to the polarized charge transfer between the fluorene donor and the cationic Ir(III) complex acceptors under an applied field. Furthermore, through the modification of the ligand structures of the Ir(III) complexes, the resulting polymers **P25** and **P26** also showed excellent memory behavior with different threshold voltage and current at the conductive state [33].

CPs containing Pt(II) complexes have also been fabricated in resistor memory devices with ITO/polymer/Al sandwiched structure [67,68]. Conjugated polyfluorene and polycarbazole with Pt(II) complexes in the side chain (**P27** and **P28**, respectively) exhibited excellent flash memory behaviors with a high ON/OFF current ratio and excellent stability with repetitive read cycles (10^7^) [67]. According to the redox properties and theoretical calculation results, the memory mechanism can be attributed to the formation and dissociation of a charge transfer state induced by negative and positive voltages, respectively. When applying an electric field over the threshold voltage, charge transfer from the polymer main chain to the side chain Pt(II) complex units occurs and switches the device to the ON state. According to the stable charge transfer complex, the ON state was still maintained even after the driving power was turned off. However, the device can be returned to the original OFF state as a reverse bias voltage is applied thus dissociating the charge transfer state. In addition, the main chain structures had significant influence on the threshold voltages. The threshold voltages of the **P28**-based device were lower than that of the **P27**-based device due to the lower oxidation potential of polycarbazole (0.43 V) than polyfluorene (0.96 V), resulting in a lower energy barrier between the work function of the ITO anode and the HOMO level of **P28** as well as an easier charge transfer compared to that of **P27**.

## 6. Flexible CP-Based Memory Devices

Flexible polymer memory devices were also demonstrated by using CPs as active layers as in other organic electronics [69]. Ueda, Liu, and Chen fabricated a typical memory device based on a flexible polyethylene terephthalate (PET) substrate [47,54], which showed a highly stable nonvolatile memory behavior even after bending up to 1000 bending cycles at a radius curvature of 5 mm. Also, the flexible memory device reported by our group [70] was tested under severe bending at various curvature radii of 11, 9, 7, and 5 mm, respectively, showing no crack or deform upon bending. The reliable and reproducible switching memory behavior of CP film in the device can also be obtained under mechanical bending stress. Similar to other organic electronics, such as organic transistors, organic photovoltaics, etc., the development of device fabrication for practical application is well underway, and the performance of polymer memory devices can be further improved by optimizing the associated processing parameters. Therefore, there is still ample opportunity for improving the electroactive materials and polymer memory devices.

## 7. Conclusions and Perspectives

Conjugated polymers for memory devices is an emerging area of intense research interest as it encompasses low cost, high mechanical strength, facile processability, and high-density data storage. This review summarized the most widely studied mechanisms in CP resistive memory devices, such as charge transfer, space charge traps, and filament conduction. Further refinements in structural design and preparation methods, enhancement in device fabrication, measurement, characterization, and integration techniques, are essential to advance polymeric memory technology.
